# Oriented immobilization of basic fibroblast growth factor: Bioengineered surface design for the expansion of human mesenchymal stromal cells

**DOI:** 10.1038/s41598-020-65572-2

**Published:** 2020-05-29

**Authors:** Ajay Shakya, Eiji Imado, Phuong Kim Nguyen, Tamamo Matsuyama, Kotaro Horimoto, Isao Hirata, Koichi Kato

**Affiliations:** 1 Department of Biomaterials, Graduate School of Biomedical and Health Sciences, Hiroshima University, Hiroshima, Japan; 2 Faculty of Odonto-Stomatology, Ho Chi Minh City University of Medicine and Pharmacy, Ho Chi Minh, Vietnam

**Keywords:** Biomaterials - cells, Stem-cell biotechnology

## Abstract

*E. coli* expressed recombinant basic fibroblast growth factor (bFGF) with histidine-tag (bFGF-His) was immobilized onto the surface of a glass plate modified with a Ni(II)-chelated alkanethiol monolayer. The immobilization is expected to take place through the coordination between Ni(II) and His-tag. The bFGF-immobilized surface was exposed to citrate buffer solution to refold *in situ* the surface-immobilized bFGF. The secondary structure of immobilized bFGF-His was analyzed by solid-phase circular dichroism (CD) spectroscopy. Immortalized human mesenchymal stromal cells (hMSCs) were cultured on the bFGF-His-immobilized surface to examine their proliferation. CD spectroscopy revealed that the immobilized bFGF initially exhibited secondary structure rich in α-helix and that the spectrum was gradually transformed to exhibit the formation of β-strands upon exposure to citrate buffer solution, approaching to the spectrum of native bFGF. The rate of hMSC proliferation was 1.2-fold higher on the bFGF-immobilized surface treated with *in situ* citrate buffer, compared to the polystyrene surface. The immobilized bFGF-His treated *in situ* with citrate buffer solution seemed to be biologically active because its secondary structure approached its native state. This was well demonstrated by the cell culture experiments. From these results we conclude that immobilization of bFGF on the culture substrate serves to enhance proliferation of hMSCs.

## Introduction

Human mesenchymal stromal cells (hMSCs) are highly desirable for applications in regenerative medicine due to their accessibility, ease of isolation, *in vitro* expansion capacities, and multi-lineage differentiation potentials. In addition, clinical trials with hMSCs so far have shown no adverse effects^1–7^. However, a poor availability of hMSCs and the requirement of a large number of cells for clinical applications warrant a huge expansion of hMSCs by *in vitro* culture^8,9^. In addition, premature senescence occurs in repeated passages with the conventional culture method^10^. Therefore, an efficient technique that addresses these problems is highly required and this is a reason why we carried out the present study.

Basic fibroblast growth factor (bFGF) is a potent mitogen for hMSCs and is routinely added in a culture medium to maintain the stemness and delay the senescence of hMSCs^11–13^. Studies reported that hMSCs expanded in the presence of bFGF exhibited faster proliferation rates than in the absence^11,14^. The activity of bFGF is dependent on its binding to one of the four FGF receptors, including FGFR1, FGFR2, FGFR3, and FGFR4, with FGFR1 being particularly important for the control of hMSC phenotype^15–18^.

In this study, attempts were made to develop a novel culture substrate for an efficient expansion of hMSCs. Our strategy is to immobilize mitogenic bFGF on the surface of a culture substrate. This is based on our hypothesis that immobilized bFGF serves to selectively capture FGFR-expressing hMSCs through the specific binding of immobilized bFGF with FGFR. This ligand binding is expected to activate FGFR signaling, leading to the promotion of cell proliferation, while maintaining the stemness and delaying senescence. A similar strategy with surface-immobilized bFGF^19^ or epidermal growth factor (EGF)^19–22^ has been proven to be feasible for efficiently expanding neural stem/progenitor cells.

According to our previous study^23^, the surface immobilization of growth factor can be suitably achieved through the chelate linkage formed between surface-bound nickel ions and a hexahistidine peptide fused with the terminus of growth factor. Such an immobilization chemistry provides structural integrity and firm attachment of the growth factor on the surface, thus, helping in the efficient expansion of cells with specific receptors. As in the previous study, we immobilized hexahistidine-tagged bFGF (bFGF-His) onto the surface of a glass-based substrate that had been modified with nickel ions.

One of the most critical aspects for biomaterials modified with bFGF may be how the protein can be integrated in the bioactive form since the three-dimensional structure of bFGF is known to be relatively unstable^24,25^. Therefore, we paid special attention to the pH of a medium that dissolves bFGF-His and a liquid phase around the immobilized bFGF-His, with the hope to properly refold the immobilized protein *in situ* at the surface. We aimed to demonstrate that hMSCs can effectively proliferate on a substrate with immobilized bFGF-His in the active form, while holding potentials to differentiate into osteogenic, chondrogenic, and adipogenic lineages.

## Materials and methods

### Expression and purification of bFGF-His

bFGF-His-coding plasmid DNA (pET22b-bFGF-His) was previously constructed^19^. The amino acid sequence of bFGF-His is shown in Fig. S1. *Escherichia coli* [*E. coli*; BL21-CodonPlus(DE3)-RIPL competent cells, Agilent Technologies, Cedar Creek, TX] transformed with pET22b-bFGF-His was cultured in terrific broth medium. In the exponential growth phase, isopropyl β-D-1-thiogalactopyranoside (Novagen; 1 mM) was added to induce the expression of bFGF-His and further cultured for 3 h. Cells were then collected by centrifugation and solubilized with BugBuster protein extraction reagent (EMD Millipore Corp, Billerica, MA) added with deoxyribonuclease (Benzonase; Merck KGaA, Darmstadt, Germany) and lysozyme. The expressed protein was purified under non-denaturing conditions over a Ni-chelated affinity column (His Trap HP; GE Healthcare Bio-Science Corp., Piscataway, NJ). The step-wise dialysis of purified bFGF-His was performed using buffer solutions shown in Table S1. After the three initial steps of dialysis, bFGF-His was further dialyzed against different buffer solutions [phosphate buffered saline (PBS) of pH 7 or citrate buffer of pH 5] at the final dialysis step to obtain three different samples: bFGF-His-7, bFGF-His-5, and bFGF-His-5/7. The bFGF-His solutions were sterilized by filtration and stored at –80 °C until use.

The concentration of protein solution was determined using Micro BCA Kit (Thermo, Waltham, MA). The purity of the protein was assessed by sodium dodecylsulfate-polyacrylamide gel electrophoresis (SDS-PAGE). Secondary structural of the protein was analyzed by circular dichroism (CD) spectroscopy. Biological activity was assessed using immortal human mesenchymal stromal cells (hMSCs, UE6E7T-3, JCRB Cell Bank, Osaka, Japan). The cells had been derived from bone marrow and immortalized by being infected with recombinant retroviruses expressing the E6, E7, and hTERT. The protocols of these analyses are provided in the Supplementary Information.

### Oriented immobilization of bFGF-His onto glass surface

bFGF-His was immobilized onto the surface of glass plates by the previously reported method with slight modifications^26^. In brief, a self-assembled monolayer (SAM) of 11-mercapto-1-undecanoic acid (COOH-thiol) was formed on the surface of a glass plate (15 mm × 15 mm × 1 mm; Arteglass Associates Co., Kyoto, Japan) coated with a thin gold layer. In order to prepare surfaces with a mixed SAM, 11-mercapto-1-undecanol (OH-thiol) was mixed with COOH-thiol at various compositions. Carboxylic acids present at the outermost surface was activated with *N*-hydroxysuccinimide (NHS) and 1-ethyl-3-(3-dimethylaminopropyl)carbodiimide and then reacted with *N*-(5-amino-1-carboxypentyl) iminodiacetic acid (NTA; Dojindo Laboratories, Kumamoto, Japan). Subsequently, the NTA introduced was then chelated with Ni(II) ions. After disinfection with 70% ethanol solution, the surface was exposed to bFGF-His solution in PBS (2 µg/mL) for 1 h at room temperature to allow for the immobilization of bFGF-His through chelation between surface bound Ni(II) and the histidine tag (Fig. S2). To prepare control samples on which bFGF-His was covalently immobilized, the surface of SAM with NHS active ester was exposed to bFGF-His-7 solution for 1 h at room temperature to allow for the formation of an amide linkage between carboxylic acids on the SAM and amines contained in bFGF-His. The second control sample was prepared by exposing the surface of COOH-SAM to bFGF-His-7 solution to allow for physical adsorption of bFGF-His-7 directly onto the SAM surface. The third control sample was prepared by physically adsorbing bFGF-His-7 onto the surface of NTA-derivatized SAM without Ni(II) chelation. The surface with COOH-thiol SAM without bFGF-His-7 immobilization was considered as a negative control. We further immobilized bFGF-His-7 by chelation onto the surfaces prepared using the mixed SAM of different COOH-thiol contents obtained by adding 11-mercapto-1-undecanol (OH-thiol).

An *in situ* citrate buffer treatment of the immobilized bFGF-His-7 was performed. For this, bFGF-His-7 immobilized on Ni(II) chelated surface was exposed to citrate buffer solution (20 mM, pH 5, 4 °C) for various duration ranging from 5 to 120 min, and then rinsed twice with PBS.

### Surface plasmon resonance (SPR) analysis

SPR analysis detects the presence of immobilized proteins on metal surface. The adsorption of bFGF-His to NTA-Ni(II) chelated substrate was analyzed in real-time using a home-made SPR sensor^27^. The protocol of the procedure is explained in Supplementary Information.

### Surface density of immobilized bFGF-His

The density of bFGF-His immobilized onto glass surface was determined using a Micro BCA protein assay kit (Thermo Fisher Scientific, Waltham, MA). The glass plate with immobilized bFGF-His-7 was placed in a well of a 24-well plate. A micro BCA reaction mixture was pipetted onto the glass plate and maintained at 37 °C for 2 h with mild shaking to allow for coloring reaction. The absorbance of the resultant solution was measured at 562 nm using a spectrophotometer (Varioskan Flash, Thermo Fisher Scientific). The surface density of immobilized bFGF-His-7 was determined using a standard curve obtained with bovine serum albumin (BSA) solutions.

### Assessment of cell proliferation

Cell proliferation was assessed on the surface of bFGF-His immobilized substrates. Glass plates (15 mm × 15 mm × 1 mm) with immobilized bFGF-His were placed in each well of a 24-well polystyrene culture plate. The hMSCs were obtained at passage twenty-four and experiments were carried out not exceeding passage twenty-eight. hMSCs were seeded onto these plates at a density of 5000 cell/cm^2^ and cultured in Dulbecco’s modified eagle’s medium (DMEM; Sigma-Aldrich, St. Louis, MO) containing 10% of Hyclone fetal bovine serum (Life Scientific, South Logan, UT), 2 mM L-glutamine (Gibco), 100 U/mL of penicillin (Sigma-Aldrich), and 100 μg/mL of streptomycin (Sigma-Aldrich) with medium exchange every alternate day. An increase in cell number was determined using Cell Counting Kit-8 (CCK-8, Dojindo Laboratories, Kumamoto, Japan) following the manufacturer’s instructions. As a control, hMSCs were cultured on a commercially available 24-well polystyrene culture plate in the presence of 1 ng/mL bFGF (Thermo Fisher Scientific). Cells were also manually counted on the phase contrast images using ImageJ 1.52 v (National Institute of Health, Bethesda, MD). The average time for the hMSCs to reach 100% confluence was 8–9 days both on 10 cm polystyrene dish and on bFGF-His immobilized substrates. During the standardization of the protocol, majority of the proliferation studies were conducted at an end point of 6 days in order to capture the cell proliferation in the logarithmic phase, as well as to avoid the effects of native ECM proteins secreted by the hMSCs during proliferation. However, upon standardization of the protocol, a detailed proliferation data at earlier time points were obtained to better understand the growth kinetics of the cells on the various substrates (bFGF-His immobilized substrates and a polystyrene dish).

### Flow cytometry

hMSCs were cultured under DMEM containing 10% FBS in a polystyrene dish. Upon reaching 80% confluence, hMSCs were harvested using a cell scrapper, starved for bFGF by incubation in a serum-free medium for 2–3 h, then blocked with 1% BSA to prevent non-specific adsorption of antibody. The cells were reacted with antibody against human FGFR1 (1:50, rabbit polyclonal; Thermo Fisher Scientific) for 1 h on ice, followed by washing with PBS containing 1% BSA. Afterward, the cells were reacted with secondary antibody (Alexa Fluor 488 anti-rabbit IgG antibody; Sigma-Aldrich) for 45 min on ice and washed with PBS containing 1% BSA. The cells were analyzed using a BD FACSCalibur flow cytometer (BD Biosciences) equipped with a 488-nm diode laser. A histogram was generated with a data from approximately 10,000 counts. Cells exposed only to secondary antibody were used as negative controls.

### Structural characterization of immobilized bFGF-His

Quartz plates (44 mm × 9 mm × 0.5 mm) were immersed in 10 µg/mL (3-aminopropyl)triethoxysilane solution for 1 h at room temperature to introduce primary amines onto the surface. Then, the plate was immersed in 0.1 mM *N*-[5-(4-isothiocyanatobenzyl) amido-1-carboxypentyl]iminodiacetic acid (isothiocyanobenzyl-NTA, Dojindo Laboratories) solution in bicine buffer for 1 h at 37 °C to allow for the reaction between the amine and the isothiocyanate. The NTA thus introduced was then used to immobilize bFGF-His on the surface by Ni(II)-mediated chelation in a similar manner to the case with the gold-coated glass plate as described earlier. To measure CD spectrum, 18 quartz plates with immobilized bFGF-His-7 were stacked and inserted into a quartz cuvette filled with PBS (pH 7.4). A CD spectrum was recorded at 20 °C using J-805 CD spectrometer (JASCO Corp., Tokyo, Japan) at an accumulation of 16. In the measurement, ultraviolet light was passed at a light angle (90 degree) normal to the quartz surfaces. After the initial measurement, the quartz plates with immobilized bFGF-His-7 were immersed in citrate buffer (20 mM, pH 5, 4 °C) for 5, 10, 30, 60, or 120 min at room temperature followed by recording CD spectra.

### Differentiation culture

MSCs expanded on bFGF-His-immobilized substrates were recovered by trypsinization and assessed for their osteogenic, chondrogenic, and adipogenic differentiation potentials according to the methods reported by other groups^4,11^ with some modifications. A detailed protocol of specific lineage differentiation is provided in Supplementary Information.

### Statistical analysis

Data are expressed as the means ± standard deviation (SD). Student’s t-tests were performed to compare means between the test sample(s) and control sample. A one-way ANOVA followed by Dunnett’s post-test was performed using IBM SPSS Statistics (IBM Corp., Armonk, NY). Statistical significance was set at *p* ≤ 0.05. Staining were performed at least triplicate.

## Results

### Preparation of bFGF-His

bFGF-His was expressed in bacteria which were newly transformed with the previously-constructed plasmid DNA^19^ and subjected to step-wise dialysis under conditions shown in Supplementary Table S1. We adopted three different buffer compositions during the final dialysis step and obtained three types of bFGF-His: bFGF-His-7, bFGF-His-5, and bFGF-His-5/7. As shown in Fig. S3, the SDS-PAGE analysis of three types of bFGF-His showed clear single bands at approximately 18 kDa, in agreement with the molecular weight predicted for bFGF-His. These results suggest that purified proteins of a correct amino acid sequence were reproducibly obtained using the new transformants.

**Figure 1:**
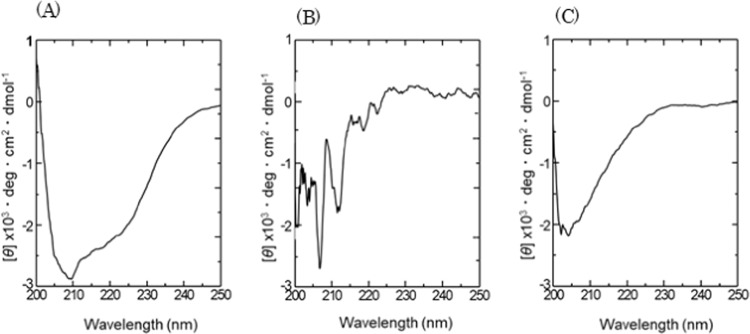
Far-ultraviolet CD spectra of bFGF-His in solution. (**A**) bFGF-His-7, (**B**) bFGF-His-5, and, (**C**) bFGF-His-5/7. *θ*: mean residue molar ellipticity.

Figure 1 shows the CD spectra of the three types of bFGF-His in solution. The spectrum of bFGF-His-7 (Fig. 1A) appears as that of proteins rich in α-helix, with strong negative Cotton effect at a wavelength of around 208 and 222 nm^28^. On the other hand, bFGF-His-5 (Fig. 1B) and bFGF-His-5/7 (Fig. 1C) gave spectra that had less contribution from α-helix at 208 and 222 nm and were similar to that of native bFGF previously reported^19^. These findings suggest that the bFGF domain in bFGF-His-5 and bFGF-His-5/7 partly refolded into the proper structure similar to native bFGF. In contrast, the bFGF domain in bFGF-His-7 is rich in α-helix, which is not in accordance with the structure reported for native bFGF^19^.

**Figure 2:**
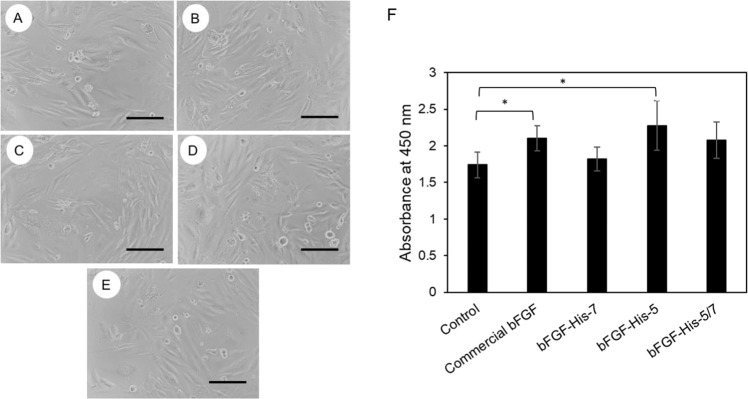
The result of cell proliferation assays performed on the polystyrene dishes. (**A–E**) Phase contrast images of hMSCs cultured on the polystyrene dishes for 6 days. hMSCs were cultured in polystyrene dishes for 6 days in a medium containing commercial bFGF (**B**), bFGF-His-7 (**C**), bFGF-His-5 (**D**), and bFGF-His-5/7 (**E**). A control experiment was conducted without growth factor (**A**). Scale bar: 200 µm. (**F**) The results of hMSCs proliferation assays on the polystyrene plates. Data are expressed as the mean ± standard deviation for n = 8. Asterisks represent statistical significance compared with control (Dunnett’s post-test, *p* < 0.05).

In order to assess the biological activity of bFGF-His, hMSCs were cultured in a medium added with one of these three proteins. As shown in Fig. 2, the addition of bFGF-His-5 resulted in the largest number of cells 6 days after cell seeding, followed by bFGF-His-5/7. On the other hand, the smallest number of cells was seen with bFGF-His-7, similar to the level when no bFGF was added. A one-way ANOVA suggests statistically significant differences according to cell proliferation conditions (p < 0.05). A Dunnett’s post test revealed significant pairwise differences between control and commercial bFGF and between control and bFGF-His-5. These results suggest that bFGF-His-5 and bFGF-His-5/7 hold biological activity, whereas bFGF-His-7 does not. This finding is consistent with our observation that the bFGF domain in the former two proteins has a proper structure and that bFGF-His-7 incorrectly refolded into the α-helix rich form.

### Surface immobilization of bFGF-His

A SPR sensorgram recorded during the exposure of the Ni(II)-carrying surface to the solution of bFGF-His-7 (Fig. S4). The SPR angle shift increased with the time of bFGF-His-7 circulation, and reached 180 mDA within 10 min. The SPR angle did not considerably decrease when the circulating solution was switched to PBS, indicating that bFGF-His-7 stably bound to the Ni(II)-carrying surface. According to the angle shift observed after 20 min circulation of PBS, the surface density of bFGF-His-7 was determined to be 0.11 µg/cm^2^ ^27^. On the other hand, when the microBCA protein assay was performed, the surface density of bFGF-His-7 was determined to be 0.53 ± 0.9 µg/cm^2^. The observed discrepancy between the results obtained by the two methods may be primarily attributed to the limited accuracy of the microBCA method under the condition adopted in this assay.

### hMSC culture on the bFGF-His-immobilized surfaces

hMSCs were cultured on different substrates prepared by various methods using bFGF-His-7. The adhesion and proliferation of hMSCs on these substrates were examined. At 24 h after cell seeding, it was observed that comparatively the larger number of cells adhered onto the surface lacking bFGF-His-7 (Fig. S5). The cells continued to proliferate on all the surfaces. The largest cell number after 6 days was observed on the surface onto which bFGF-His-7 was immobilized by coordinating to the Ni(II)-NTA (Fig. S5E).

**Figure 3:**
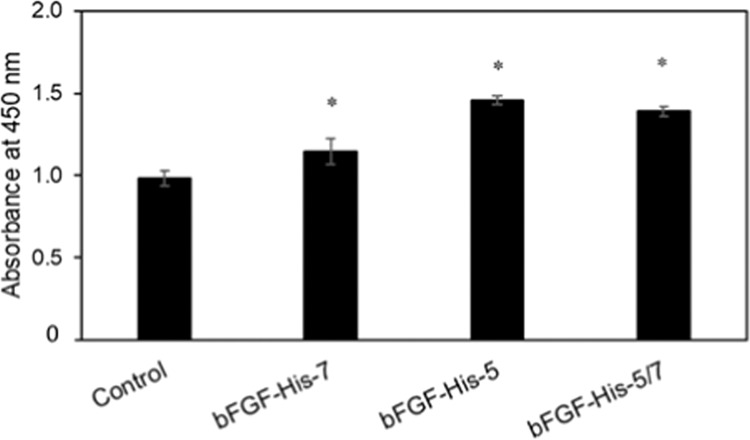
The result of hMSC proliferation assays performed on the surfaces onto which three different types of bFGF-His were immobilized by chelation. As a control, hMSCs were cultured on a Ni(II) chelated surface lacking immobilized bFGF-His. The assays were preformed 6 days after cell seeding. Data are expressed as the mean ± standard deviation for n = 4. Asterisks indicate significant difference compared with control (Student’s t-test, *p* < 0.05).

hMSCs were also cultured on the substrates prepared by immobilizing either of three types of bFGF-His. The result of the cell proliferation assay on day 6 (Fig. 3) showed that, among the surface with three types of bFGF-His, largest cell number was observed on bFGF-His-5 immobilized substrate, and minimal number on the substrate with bFGF-His-7.

### Expression of FGFR1

The result of flow cytometry (Fig. S6) conducted using anti-FGFR1 antibody showed that the content of FGFR1-positive cells in the total cells was 74.2%. This result indicates that FGFR1-positive cells were present on these cells cultured thorough the standard culture protocol on polystyrene plates.

### COOH-thiol composition

**Figure 4:**
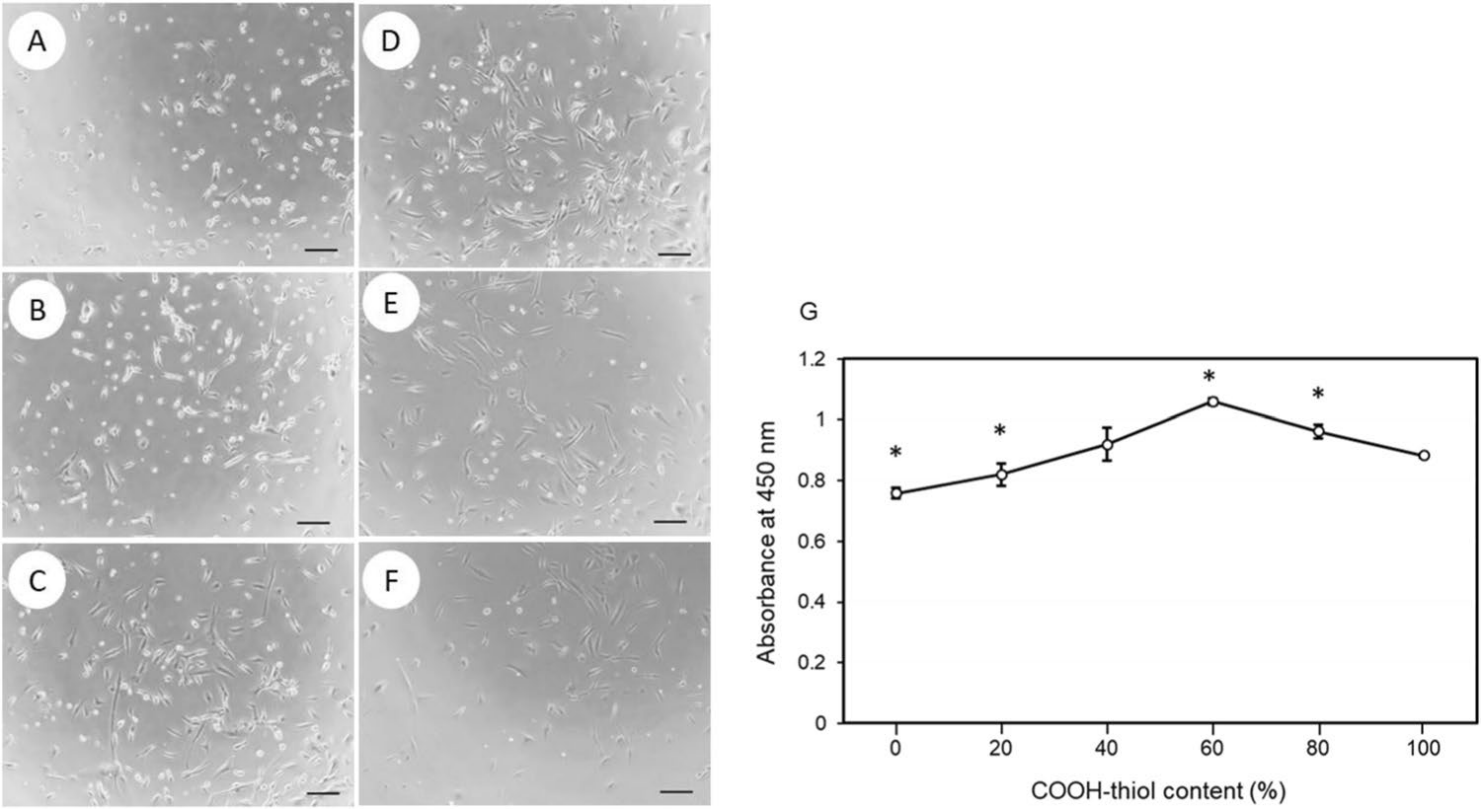
The results of adhesion and proliferation assays on the bFGF-His-7 immobilized surfaces prepared using SAMs with different alkanethiol compositions. (**A–F**) Phase contrast images of hMSCs 24 h after cell seeding. A COOH-thiol content in the SAM: (**A**) 0%, (**B**) 20%, (**C**) 40%, (**D**) 60%, (**E**) 80%, and (**F**) 100%. Scale bar: 200 µm. (**G**) The result of hMSC proliferation assays plotted as a function of COOH-thiol contents. The assays were conducted 6 day after cell seeding. Data are expressed as the mean ± standard deviation for n = 4. Asterisks indicate significance difference compared to the case with 100% COOH-thiol (Student’s t-test, *p* < 0.05).

The effect of alkanethiol composition in SAM was studied in terms of cell proliferation. Cells were cultured on bFGF-His-7 immobilized substrates prepared using mixed SAMs of different COOH-thiol contents (Fig. 4). When observed 24 h after initial seeding, minimal cell adhesion was observed on the substrate with 0% COOH-thiol content. Cells on this substrate appeared rounded and disintegrated. With the passing days, cells continued to proliferate on all the surfaces, reaching largest cell number on the substrate prepared using 60% COOH-thiol content (Fig. 4G).

### *In situ* activation of immobilized bFGF-His

As per the comparative study of different bFGF formats (Fig. 3), the bFGF-His-7 performed poorly of all three types of bFGF-His immobilized surfaces. For further studies, we focused on the bFGF-His-7 to examine the possibility to fold its structure into the proper form and make it biologically active. As evident from the results described in the preceding sections, the secondary structure and biological activity of bFGF-His greatly depend on the conditions of buffer solutions. This finding led us to hypothesize that bFGF-His-7 in the denatured state might be able to refold into the bioactive form by the citrate buffer treatment, even after immobilization onto the surface of a glass-based substrate.

To test our hypothesis, we immobilized bFGF-His-7 by chelation between the surface bound Ni(II) and the histidine tag on 60% COOH-thiol substrate. The immobilized bFGF-His-7 was subjected to *in situ* citrate buffer treatment (20 mM, pH 5, 4 °C) for 60 min at room temperature, following which hMSCs were cultured on those substrates. On day 6, more rapid cell proliferation was observed on the bFGF-His-7 immobilized substrate which was exposed *in situ* to citrate buffer, compared to the bFGF-His-7 immobilized substrate without *in situ* citrate buffer treatment (Fig. S7).

**Figure 5:**
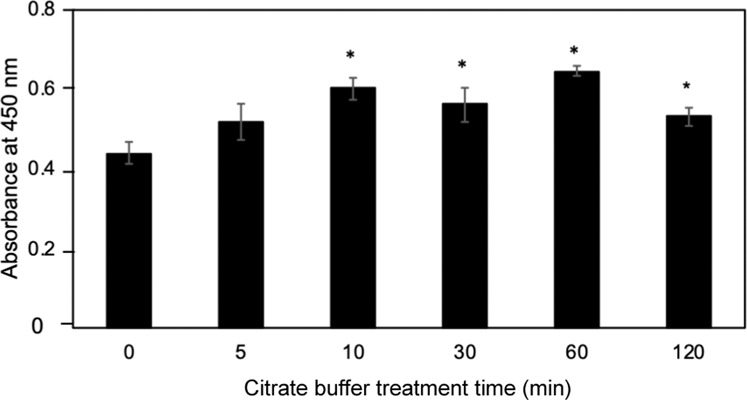
The proliferation of hMSCs on the bFGF-His-7 immobilized surfaces treated with citrate buffer solution for different time periods. The hMSC proliferation assays were performed 6 days after seeding. Data are expressed as the mean ± standard deviation for n = 4. Student’s t-test was performed to analyze significance in difference compared with the control sample with no citrate buffer treatment (*p < 0.05).

The effect of duration of *in situ* citrate buffer treatment was further studied in detail. The immobilized bFGF-His-7 was subjected to *in situ* citrate buffer treatment (20 mM, pH 5, 4 °C) for different time duration (0 min – 120 min). On par with the previous finding (Fig. S7), the largest cell number was observed by 60-min *in situ* citrate buffer treatment and the smallest by no *in situ* activation (Fig. 5).

**Figure 6:**
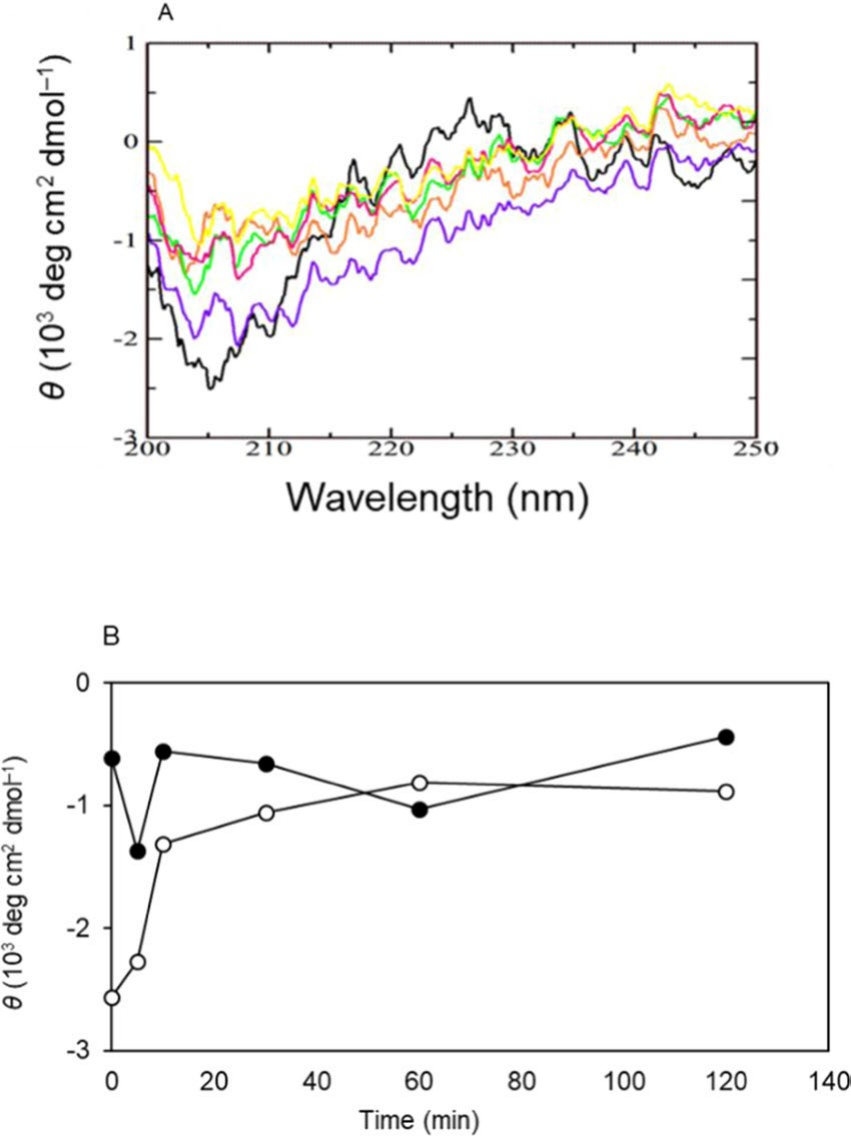
Secondary structural changes of immobilized bFGF-His-7 during the *in situ* treatment with citrate buffer. (**A**) Far-ultraviolet CD spectra of immobilized bFGF-His-7 after the citrate buffer treatment for (black) 0, (purple) 5, (red) 10, (green) 30, (orange) 60, and (yellow) 120 min. (**B**) Mean residue molar ellipticity at (open circle) 208 and (closed circle) 216 nm as a function of the duration of the citrate buffer treatment.

Once it was apparent that 60-min *in situ* citrate buffer treatment significantly improved the biological activity of originally inactive bFGF-His-7, we further tried to gain insights into the secondary structural changes by *in situ* citrate buffer treatment. The solid-phase CD spectroscopy analysis was performed for the secondary structure of immobilized bFGF-His-7 before and after exposure to citrate buffer solution of pH 5. The results are shown in Fig. 6A. Because of limited protein molecules present in a light path, the signal-to-noise ratio was small. However, we could still observe the negative Cotton effect at a wavelength around 208 nm for α-helix contained in bFGF-His-7 before the citrate buffer treatment. In order to monitor the time-dependent alteration of the secondary structure, mean residue molar ellipticities observed at 208 nm (α-helix) and 216 nm (β-strand) are plotted as a function of time in Fig. 6B.

The magnitude of a negative Cotton effect at 208 nm was significantly reduced within 10 min and reached the smallest negativity by 60-min citrate buffer treatment. In contrast, the magnitude of a negative Cotton effect at 216 nm slightly increased its negativity with time and reached the largest negativity 60 min after the onset of the treatment. These results suggest that immobilized bFGF-His-7 turned from the α-helix-rich form into the β-strand-containing structure at surface.

### Comparison with polystyrene culture plates

**Figure 7:**
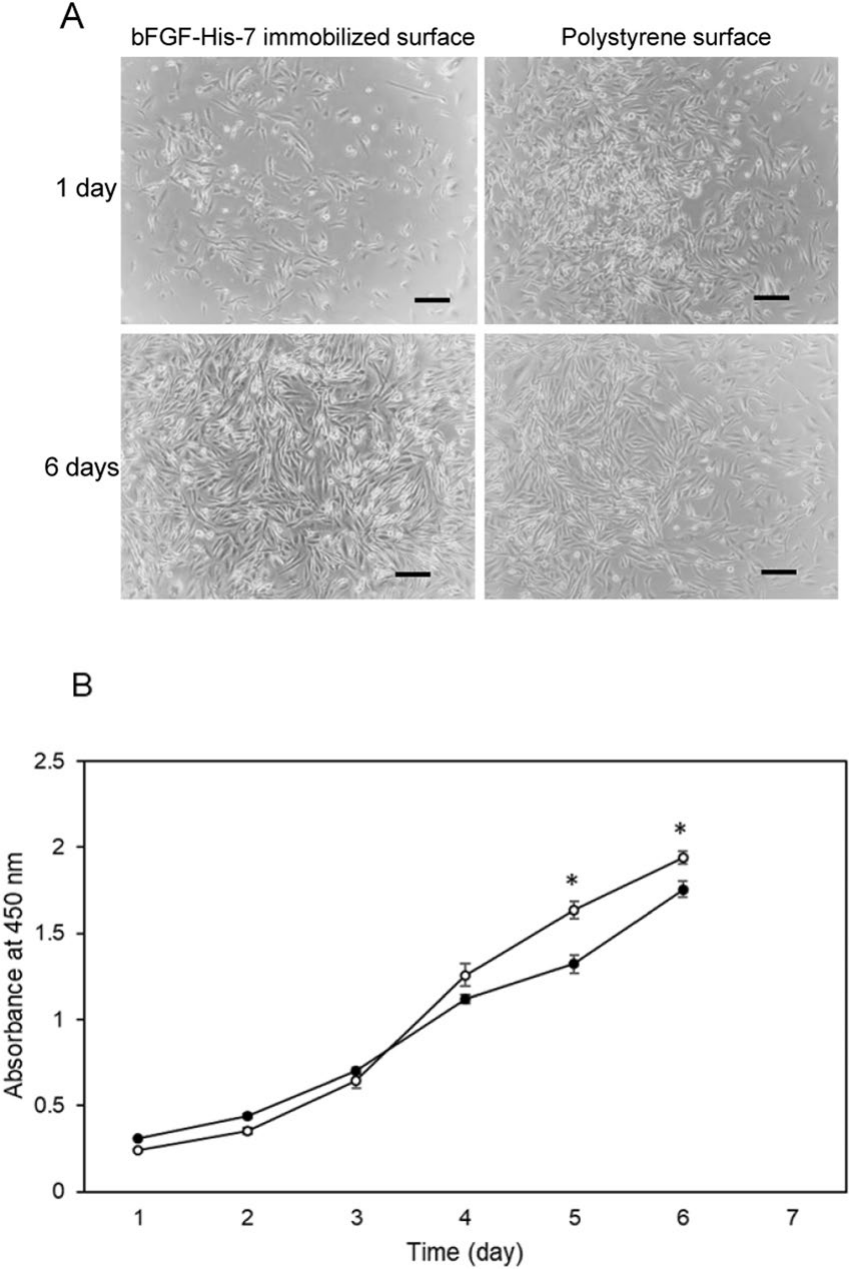
The proliferation of hMSCs on the bFGF-His-immobilized, *in situ* citrate buffer-treated surface. (**A**) Phase contrast images of hMSCs cultured on the surface of (left) the bFGF-His-immobilized, *in situ* citrate buffer-treated substrates and (right) polystyrene plates for (upper) 1 day and (bottom) 6 days. Scale bar: 200 μm. (**B**) The results of hMSCs proliferation assays on the surface of (open circle) the bFGF-His-immobilized, *in situ* citrate buffer-treated substrates and (closed circle) polystyrene plates. Data are expressed as the mean ± standard deviation for n = 4. Student’s t-test was performed to analyze significance in difference between the two conditions at the same time points (*p < 0.05).

Based on the results described above, the bFGF-His-7 immobilized substrate with 60-min *in situ* citrate buffer treatment was used in this experiment. When analyzed 24 h after seeding, the number of hMSCs adhered on the polystyrene surface was larger compared to the bFGF-His-7 immobilized surface. There may be more weakly attached cells on the polystyrene surface, which were easily detached from the surface by PBS wash. However, no obvious differences in morphology were noted among the two populations during continued culture (Fig. 7A). Cell growth on both the surfaces followed the similar trend throughout the culture period (Fig. 7B). However, a significantly higher number of cells was seen on the bFGF-His-7 immobilized substrate than the polystyrene culture plates at day 5 and day 6. A straightforward manual counting of cells was made on the phase contrast images (6 images per condition) of cells cultured on the bFGF-His immobilized substrate and polystyrene dish on day 6 using ImageJ 1.52 v. The results also revealed a significantly higher cell number on the bFGF-His-7 immobilized substrate (565 ± 48 SD cells per field of view) than on the polystyrene dish (445 ± 63 SD per field of view) (Student’s t-test, p < 0.05). These results suggest that, within the same culture duration, the large number of cells can be expanded on *in situ* citrated buffer treated, bFGF-His-7 immobilized substrates than conventional polystyrene culture plates.

### Multilineage differentiation potential

**Figure 8:**
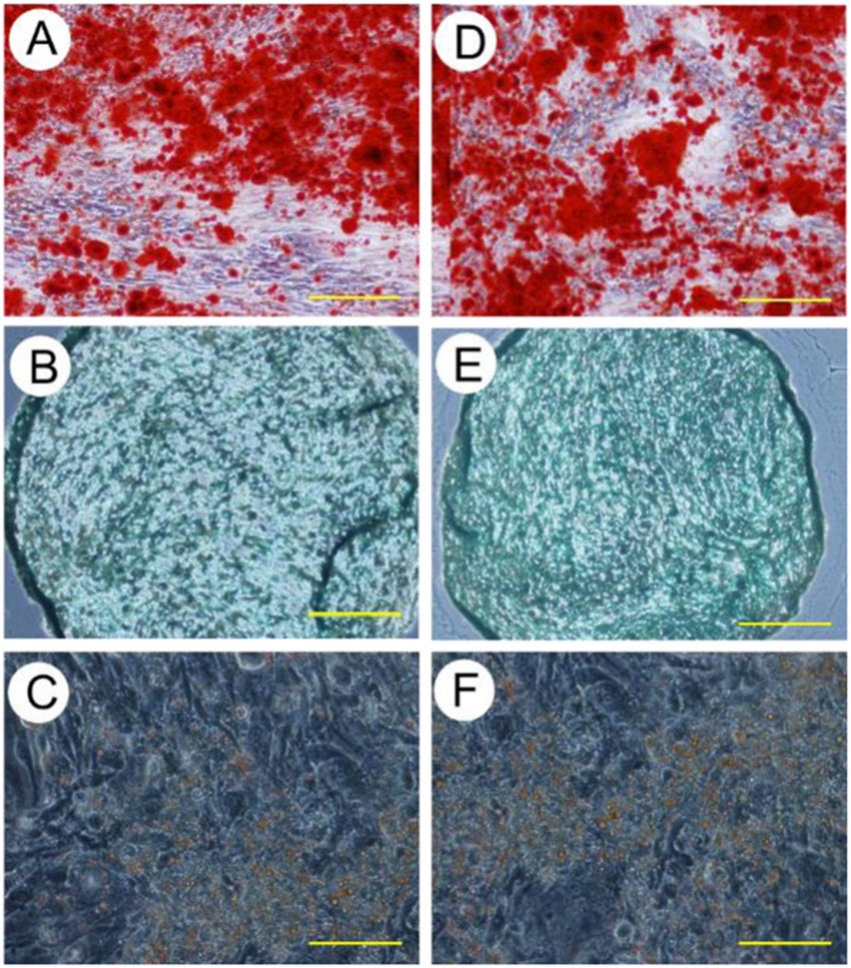
The results of differentiation assays with (**A**–**C**) hMSCs obtained from the bFGF-His-immobilized, *in situ* citrate buffer-treated substrates and (**D**–**F**) polystyrene plates. (**A**,**D**) Optical microscopic images of cells cultured in the osteogenic medium for 28 days and then stained with Alizarin red. (**B**,**E**) Optical microscopic images of cells cultured as pellets in the chondrogenic medium for 28 days and then stained with Alcian blue. (**C**,**F**) Optical microscopic images of cells cultured in the adipogenic medium for 21 days and stained with oil red O. Scale bar: 200 μm.

hMSCs obtained from the bFGF-His-immobilized surface and the conventional polystyrene plates were subjected to undergo differentiation into osteogenic, chondrogenic and adipogenic cell lineages. After 28 days in the osteogenic induction medium, calcium deposits stained with Alizarin red were found in both culture on the bFGF-His-immobilized surface and the polystyrene surface (Fig. 8A and D). In the case of chondrogenic differentiation, pellets from both cultures were stained with Alcian blue at a similar level each other (Fig. 8B and E). On the other hand, oil red O staining was used to assess the level of adipocyte differentiation^29^. After 28-day culture in the adipogenic induction medium, lipid vesicles were stained with oil red O in both the cells (Fig. 8C and F). These results suggested that cells obtained from the bFGF-His-immobilized substrate have multilineage differentiation potentials similar to the cells from the standard polystyrene plates.

## Discussion

In an attempt to develop novel culture substrates that permit efficient expansion of hMSCs, we immobilized histidine-tagged bFGF (bFGF-His) onto the surface of solid substrates. The immobilization reaction was achieved through the chelating reaction of the histidine-tag with Ni(II) ions introduced onto the surface of glass-based substrates. In this study, we demonstrated that the bFGF-His-immobilized surface provides a substrate on which hMSCs can efficiently proliferate at a level higher than the standard polystyrene surface, while preserving the multi-lineage differentiation potentials of the cells. The successful outcome can be greatly attributed to the structural integrity of the bFGF domain of the fusion protein.

It is known that the 3D structure of bFGF is relatively unstable^24,25^, partly because this protein contains no S–S linkage that would stabilize the protein structure against physicochemical perturbation. In the present study, bFGF-His was obtained from *E. coli* in the soluble form and could be extracted and purified under conditions that did not require any addition of denaturing agents such as urea. It is likely that these conditions benefitted the structural integrity of the bFGF domain. However, our results showed that buffer conditions during medium exchange by dialysis can affect the secondary structure and thus the biological activity of bFGF-His. That is why we paid special attention to the structural alternation of bFGF-His in solution and also at surface.

We found that the secondary structure (Fig. 1) and the biological activity (Fig. 2) of bFGF-His in solution drastically altered depending on buffer conditions employed at the final step of dialysis programs (Table S1). The bFGF domain exerted acidic conditions, namely pH 5, in 20 mM citric acid buffer and was found to properly refold into the structure similar to the native form of bFGF^30,31^, as evidenced by our observation that the protein rich in α-helix turned into a β-sheet-rich, native bFGF-like form when the buffer was switched from neutral PBS to citrate buffer of pH 5 (Fig. 1).

In the dialysis process, the pH and ionic strength of dialysis buffer solution are known to play a crucial role in the successful refolding of proteins^32^. The isoelectric point (pI) of protein may give us a clue about the optimal pH at which the protein is dialyzed. Research indicated that proteins with acidic pI refolded in buffers of alkaline pH and *vice versa*^33^. Since the pI represents the pH where protein does not have net charges, it also corresponds to the pH where protein has minimal solubility. Giving a pH shock to a protein by dialyzing against a buffer solution of pH distant from the pI of the protein promoted the solubilization and recovery of its bioactive form^34^. For bFGF, which is a highly basic protein with an isoelectric point at 9.6^35^, it is likely that a buffer solution of lower pH can accelerate its refolding.

The sensitivity of bFGF against buffer conditions rather provides us with an opportunity to refold the originally-denatured, immobilized bFGF-His *in situ* at surface, as we demonstrated in this study. The results of biological assays using hMSCs (see Supplementary Fig. S7 and Fig. 5) and solid-phase CD spectroscopy (Fig. 6) suggest that the immobilized bFGF-His-7 indeed refolds into the β-sheet-rich, bioactive form within 60 min. The spectra observed after the 60-min treatment with citrate buffer was in close approximation to the spectra observed previously for the immobilized bFGF-His^19^. It is generally considered that folding of a single protein molecule takes place in a millisecond time-scale^36^. However, it takes longer periods of time, namely several tens of minutes, for the immobilized bFGF-His to totally recover its secondary structure and biological activity at surface. This discrepancy may be due to the effect that segmental rearrangements are constrained by the interactions of immobilized protein molecules with substrate surfaces. Nevertheless, the possibility of activating *in situ* the immobilized bFGF-His is of advantage from a practical point of view, because we do not need to care about the denaturation of bFGF-His during the immobilization reaction and the storage of the bFGF-His-immobilized substrates.

The adhesion and proliferation of hMSCs were studied on the bFGF-His-immobilized substrates by seeding hMSCs right after immobilization of the protein. It took longer period of time (approx. 36 h) for the cells to adhere and achieve fibroblast-like appearance on the bFGF-His-immobilized surface, compared to cells seeded on polystyrene. The transient delay in cell adhesion could be partly due to reduced FGFR density on the surface of hMSCs as a result of trypsinization^37–39^. Because only FGFR-expressing hMSCs are expected to be trapped on the bFGF-His-immobilized surface through bFGF-FGFR interactions, it may take time for cells to recover FGFR on their surface.

The results of the present study show that hMSCs proliferated most effectively on the substrate with chelated bFGF-His, compared to the case with bFGF-His physically adsorbed or covalently immobilized. This is likely because the chelating method enhances the stability of immobilized protein and the structural integrity during immobilization reactions, as suggested by Nakaji-Hirabayashi *et al*. for neural stem cells adhering to EGF-His immobilized surfaces^23^. In the present study, we further observed that hMSCs adhered and proliferated even in the absence of immobilized bFGF-His (Fig. S5 and Fig. 3). This observation is probably due to the serum contained in the medium. Adhesive proteins, such as fibronectin and vitronectin in fetal bovine serum, might mediate the adhesion of cells to the culture surface through integrins^40^.

During the routine culture of hMSCs on 10 cm polystyrene dish, the culture medium was regularly supplemented with commercial recombinant bFGF (1 ng/mL) on every alternate day. Upon 80% confluence, the cells were harvested and reseeded with and without supplemented bFGF. When compared to the non-bFGF supplemented culture, initially there was no significant difference in terms of the rate of cell proliferation with and without bFGF. However, the rate of cell proliferation gradually decreased with successive passages of non-bFGF supplemented hMSCs. The rate of cell proliferation with supplemented bFGF remained unaffected. This could be the reason for the minimal difference in the improvement of cell proliferation for all the experimental groups containing bFGF compared to the non-bFGF containing control (Fig. 2 and Fig. 3). The cells for both the conditions were obtained from the same source (bFGF supplemented culture medium).

Steric repulsion effect, exhibited by triethylene glycol (TEG) at the terminus of an alkanethiol, renders surfaces resistant to the adsorption of proteins^41^. Incorporation of TEG-thiol to COOH-thiol SAMs prevented non-specific adsorption of EGF onto SAM surface, elevating the fraction of oriented EGF-His^20^. In the present study, we incorporated OH-thiol, which has a similar property to TEG-thiol. The OH-thiol is excellent in preventing nonspecific adsorption of proteins^42,43^. The highest cell proliferation was seen on the bFGF-His immobilized surface prepared from a mixed SAM of 60% COOH-thiol. The cell proliferation declined beyond 60% COOH-thiol content (Fig. 4). We speculate that excess COOH rather attracts non-specific adsorption of bFGF-His, which might cause denaturation of the protein, reducing its biological effects. Based on all the findings discussed till now, immobilizing bFGF-His-7 on mixed SAM of 60% COOH-thiol content followed by *in situ* citrate buffer activation for a duration of 60 min was taken as the optimal protocol for further cell proliferation experiments.

The proliferation of hMSCs on the surface onto which bFGF-His was immobilized as above was compared with that on the surface of a conventional polystyrene dish. It was observed that cell proliferation on bFGF-His-immobilized surface was sluggish for initial 3 days. However, from day 4 onwards, more rapid proliferation was observed on bFGF-His-immobilized surface than on polystyrene (Fig. 7). The initial sluggishness of cell growth on bFGF-His immobilized substrate could be due to the reasons discussed earlier. The number of proliferated cells was found to be 1.2 times larger on the citrate buffer-activated, bFGF-His immobilized substrate than on the standard polystyrene dish.

The cells expanded on the bFGF-His immobilized substrate retained their capacity for multipotent differentiation as they could successfully differentiate *in vitro* into osteogenic, chondrogenic, and adipogenic lineages (Fig. 8) at a similar level to the cells grown on a polystyrene plate. The *in vitro* tri-lineage differentiation has been considered as a basis for testing tissue-specific MSCs in numerous preclinical and clinical trials^4,44^. Accordingly, it may be concluded that the captured MSCs proliferated on the bFGF-His immobilized substrate proliferate faster than on polystyrene, while retaining their differentiation potentials. It can also be speculated that cells expressing FGFR are selectively captured on the bFGF-immobilized surface, whereas any cells can be captured on polystyrene surface. This assumption paves way for future studies in this direction. Further evaluation of the bFGF-His-immobilized substrates with different primary cell populations is highly recommended and will be carried out in future. It will help to broaden the scope of our culture technique. Since we used immortalized human bone marrow-derived mesenchymal stem cells (UE6E7T-3), we did not conduct any staining of senesce marker such as beta galactosidase (commonly used in *in vitro* replicative senescence studies). However, it is highly advisable to look for senescence markers while using primary cells.

## Conclusions

This study demonstrates that bFGF-His can be refolded partially into the bioactive form by dialyzing it against citrate buffer solution of pH 5. This is also the case for surface-immobilized bFGF-His. Substrates on which bFGF-His is immobilized by chelation serve to enhance the proliferation of hMSCs, while retaining their multilineage differentiation potentials.

## Supplementary information

Supplementary Information.
