# Supplementary material for: Oriented immobilization of basic fibroblast growth factor: Bioengineered surface design for the expansion of human mesenchymal stromal cells
Source: Sci Rep. 2020 May 29;10:8762. doi: 10.1038/s41598-020-65572-2 (PMC7260242; doi:10.1038/s41598-020-65572-2)
Supplement: Supplementary file 1 — Supplementary Information. [file 41598_2020_65572_MOESM1_ESM.pdf]

Supplementary Information

Oriented immobilization of basic fibroblast growth factor: Bioengineered surface design for the expansion of human mesenchymal stromal cells

Ajay Shakya[a], Eiji Imado[a], Phuong Kim Nguyen[a, b], Tamamo Matsuyama[a], Kotaro Horimoto[a], Isao Hirata[a], and Koichi Kato[a]*

[a]Department of Biomaterials, Graduate School of Biomedical and Health Sciences, Hiroshima University, Japan

[b]Faculty of Odonto-Stomatology, Ho Chi Minh University of Medicine and Pharmacy, Vietnam

***Corresponding author**

E-mail address: kokato@hiroshima-u.ac.jp

Tel: +81-82-257-5645

Fax: +81-82-257-5649

```
            10         20         30         40         50         60
  1 MAAGSITTLP ALPEDGGSGA FPPGHFKDPK RLYCKNGGFF LRIHPDGRVD GVREKSDPHI
 61 KLQLQAEERG VVSIKGVCAN RYLAMKEDGR LLASKCVTDE CFFFERLESN NYNTYRSRKY
121 TSWYVALKRT GQYKLGSKTG PGQKAILFLP MSAKSLEHHH HHH
```

Figure S1. Amino acid sequence of bFGF-His of total 163 aa. Fist 155 amino acids are for full-length bFGF followed by "LE" (underlined) coming from the plasmid and then six consecutive histidine (His-tag).

Table S1.

Buffer solutions used for step-wise dialysis. Conditions of type I, II, and III were adopted for preparing bFGF-His-5, -7, and 5/7, respectively.

Type I (bFGF-His-5)

Step	Buffer composition	pH
1	PBS, 3.75 mM glutathione, 0.375 mM glutathione, 0.2 M L-arginine	7.0
2	PBS, 3.75 mM glutathione, 0.375 mM glutathione, 0.1 M L-arginine	7.0
3	PBS, 1 mM glutathione, 0.1 mM glutathione	7.0
4	PBS	7.0
5	20 mM citrate buffer	5.0

Type II (bFGF-His-7)

Step	Buffer composition	pH
1	PBS, 3.75 mM glutathione, 0.375 mM glutathione, 0.2 M L-arginine	7.0
2	PBS, 3.75 mM glutathione, 0.375 mM glutathione, 0.1 M L-arginine	7.0
3	PBS, 1 mM glutathione, 0.1 mM glutathione	7.0
4	PBS	7.0

Type III (bFGF-His-5/7)

Step	Buffer composition	pH
1	PBS, 3.75 mM glutathione, 0.375 mM glutathione, 0.2 M L-arginine	7.0
2	PBS, 3.75 mM glutathione, 0.375 mM glutathione, 0.1 M L-arginine	7.0
3	PBS, 1 mM glutathione, 0.1 mM glutathione	7.0
4	PBS	7.0
5	20 mM citrate buffer	5.0
6	PBS	7.0

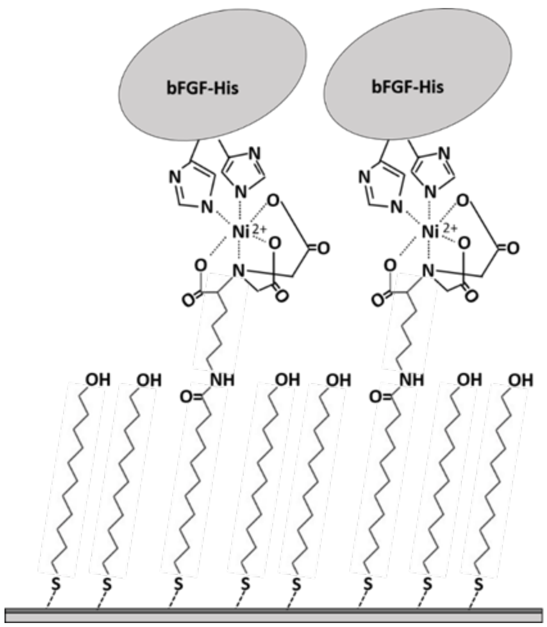

Figure S2. Schematic illustration for the bFGF-His-immobilized surface. Protein molecules are not scale.

SDS-PAGE

To investigate the molecular size and purity of bFGF-His, sodium dodecyl-sulfate-polyacrylamide gel electrophoresis (SDS-PAGE) was performed. Loading buffer (Mercaptoethanol : Laemmli sample buffer = 1 : 19) was added to protein solutions. Standard protein solution (Precision Plus Protein Standards, Bio-Rad, USA) and sample solutions were heated at 95 ^{o}C for 15 min and then cooled at 4 ^{o}C. An acrylamide gel (12%) in tris-glycine buffer (Mini-PROTEAN TGX Precast Gels, Bio-Rad, USA) was placed in an electrode chamber, and running buffer (tris/glycine/SDS buffer) was poured into inner and outer chambers. Standard protein solution and sample solutions were loaded into wells. Electrophoresis was run at 200 V until one of the small molecular dyes almost reached the bottom line of the gel. After electrophoresis, the gel was removed from a cassette and washed before being stained by Quick-CBB plus solution overnight. Next day, gel was washed, and stained bands were imaged under white light in ChemiDoc XRS+ System (Bio-Rad, USA). The result is shown in Supplementary Figure S3.

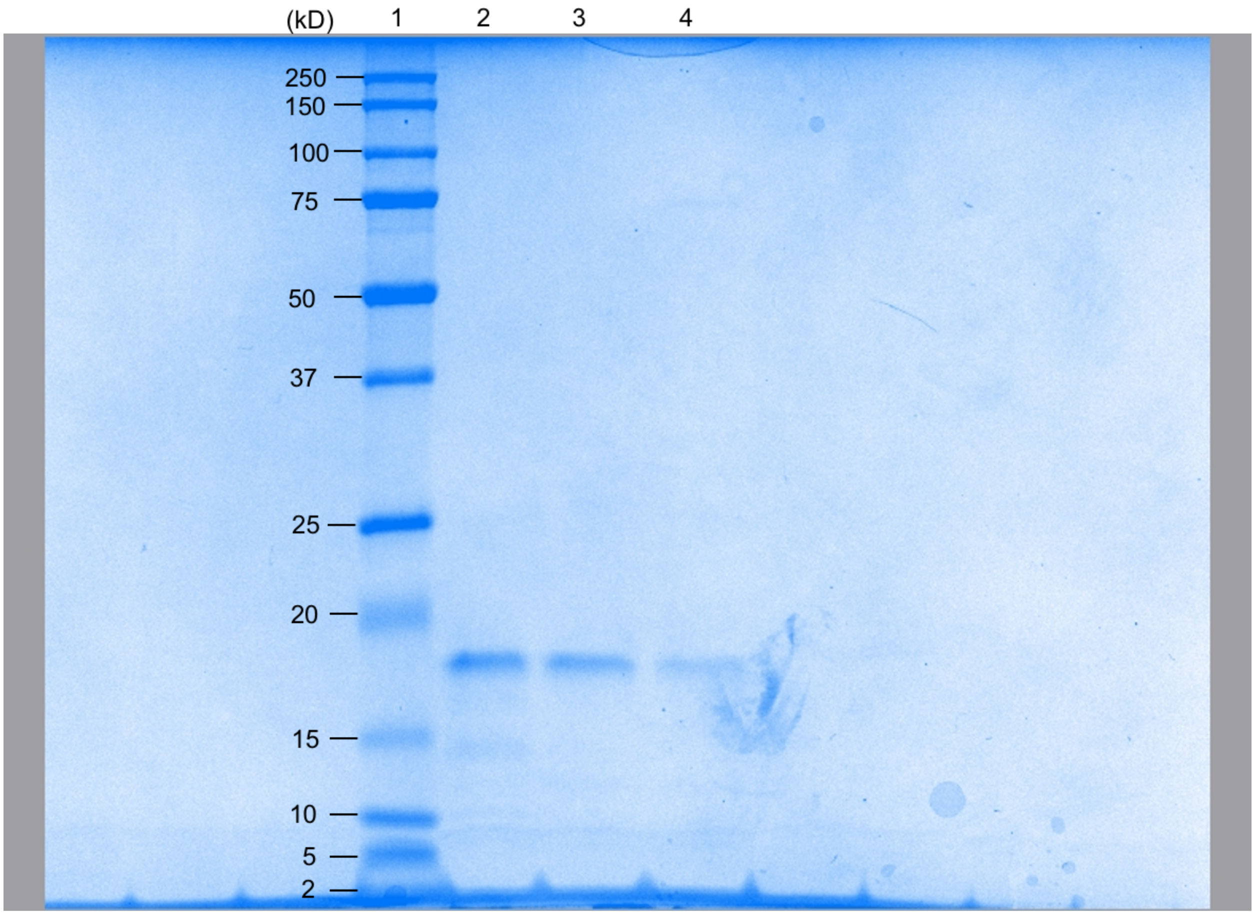

Figure S3. SDS-PAGE analysis of bFGF-His. Lane 1: Molecular weight standard; Lane 2: bFGF-His-5; Lane 3: bFGF-His-7; Lane 4: bFGF-His-5/7.

Circular dichroism (CD) spectroscopy

Structural characteristics of three types of bFGF-His in solution were analyzed by CD spectroscopy. For this, 100 µg/ml bFGF-His solution was prepared by diluting the stock solution with PBS (pH 7.4). The CD spectra were recorded with J-805 CD spectrometer (JASCO Corp., Tokyo, Japan) at 20 ^{o}C and a 1-mm path length cell. The measurement was performed at a response time of 0.5 s and a scan speed of 100 nm/min with a bandwidth of 1 nm, and an accumulation of 4.

Surface plasmon resonance analysis

A home-made surface plasmon resonance (SPR) sensor was used to analyze the adsorption of bFGF-His to NTA-Ni(II) chelated substrate in real-time.[1] The detailed elaboration of the procedure is explained elsewhere.[1--4] The surface of a BK7 glass plate (Refractive index = 1.515; Glass Dynamics LLC, Vineland, NJ) was modified with NTA and Ni(II) ions as described above. The Ni(II)-chelated plate was mounted in a home-made SPR apparatus[1] with a HeNe laser light source (632.8 nm) and a flow cell. After equilibration of the sample surface with PBS for approximately 1 min, 2 µg/mL bFGF-His-7 solution in PBS was circulated over the surface to allow for the immobilization of bFGF-His. After 20 min, the solution was switched to PBS and circulated for more 20 min. During these circulation processes, reflectance was continuously recorded with photomultiplier tubes at a constant incident angle slightly above the resonance angle. The angular shift observed before and after the circulation of bFGF-His was converted to the amount of bFGF-His bound to the surface, assuming that unit angular shift corresponds to a protein density of 1.1 mg/cm^2.[1] The result is shown in Figure S2.

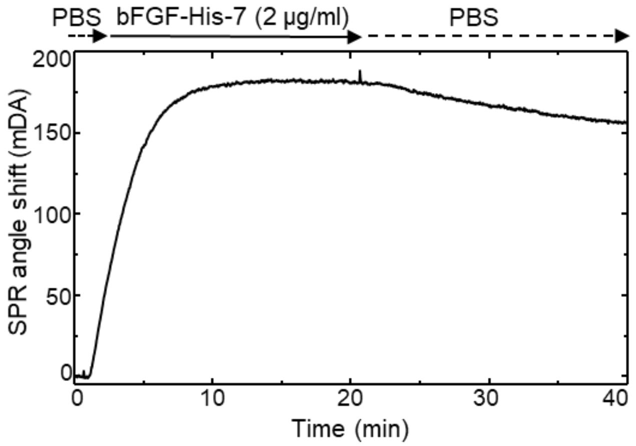

Figure S4. SPR sensorgrams recorded during the exposure of the Ni(II) ion-carrying surface to 2 µg/mL bFGF-His solution and the subsequent washing of the surface with PBS.

Biological assays

hMSCs were cultured in a 96-well polystyrene culture plate. The medium was supplemented with one of the three types of recombinant human bFGF-His (1 ng/mL). Cells cultured in the medium supplemented with commercial recombinant human bFGF (1 ng/ml; Thermo Fisher Scientific) was used as a positive control, whereas cells cultured without any supplemented bFGF in the medium was considered as a negative control. A fresh medium consisting of Dulbecco's modified eagle's medium (DMEM; Sigma-Aldrich, St. Louis, MO), 100 U/mL of penicillin (Sigma-Aldrich), 100 µg/mL of streptomycin (Sigma-Aldrich), 2 mM L-glutamine (Gibco), and 10% of Hyclone fetal bovine serum (Life Scientific, South Logan, UT) was exchanged every alternate day. Cells were periodically observed by an optical microscope to assess the level of confluence. Cell proliferation assay was performed using Cell Counting Kit-8 (CCK-8) (Dojindo Laboratories, Kumamoto, Japan). CCK-8 solution was pipetted into the well of a 96-well polystyrene culture plate, incubated for 2 h at 37 °C, and absorbance at 450 nm was measured for the resulting solution using a spectrophotometer (Varioskan Flash, Thermo Fisher Scientific).

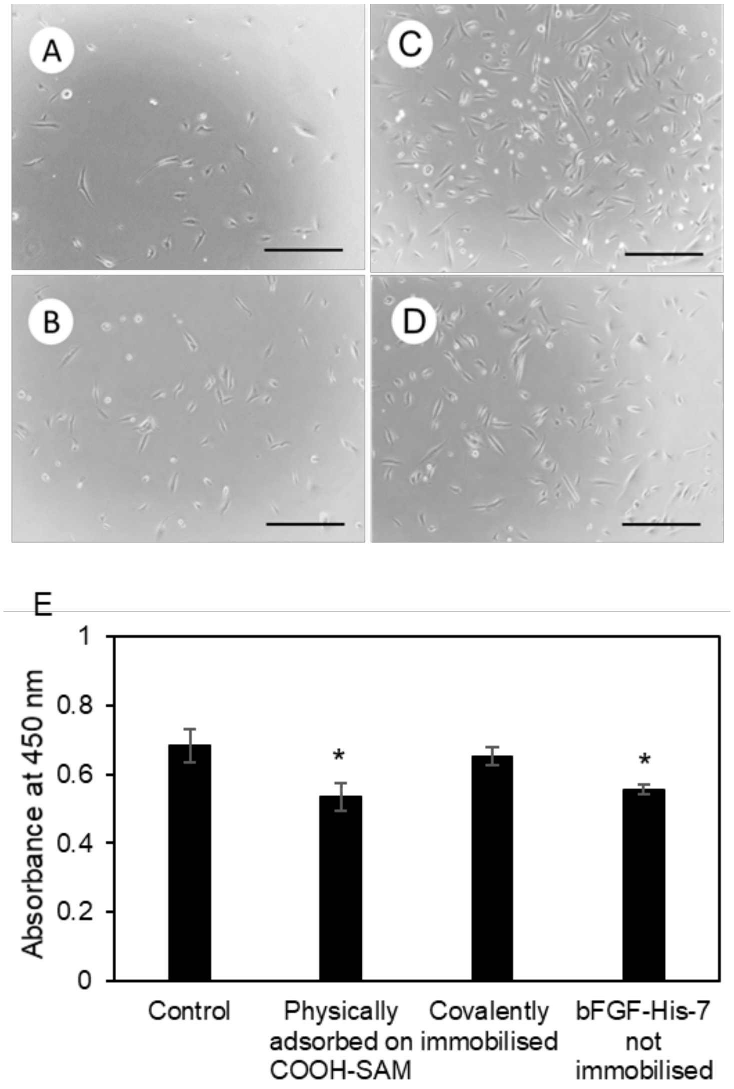

Figure S5. Adhesion and proliferation of hMSCs on the surface with adsorbed and immobilized bFGF-His. (A–D) Phase contrast images of hMSCs seeded onto various surfaces. (A) Ni(II) ion-carrying NTA-SAM with chelated bFGF-His, (B) NTA-SAM (no Ni(II)ions) with physically adsorbed bFGF-His-7, (C) COOH-SAM covalently immobilized bFGF-His, and (D) pristine SAM. Images were acquired 24 h after cell seeding. Scale bar 200 μm. (E) The result of hMSC proliferation assays performed on various surfaces 6 days after cell seeding. Data are expressed as the mean ± standard deviation for n = 4. Student's t-test was performed to analyze significance in difference compared with control (*p < 0.05).

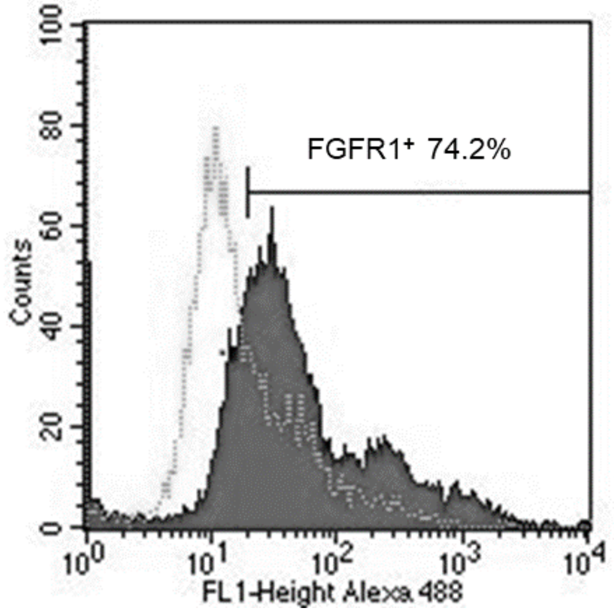

Figure S6. Flow cytometry analysis for the expression of FGFR1 on hMSCs. The area shaded in gray represent the result from cells reacted with anti-FGFR1 antibody, while the dotted curve represents the result of a control experiment in which cells were treated only with secondary antibody. The percentage of FGFR1 positive cells was determined using the intersection point.

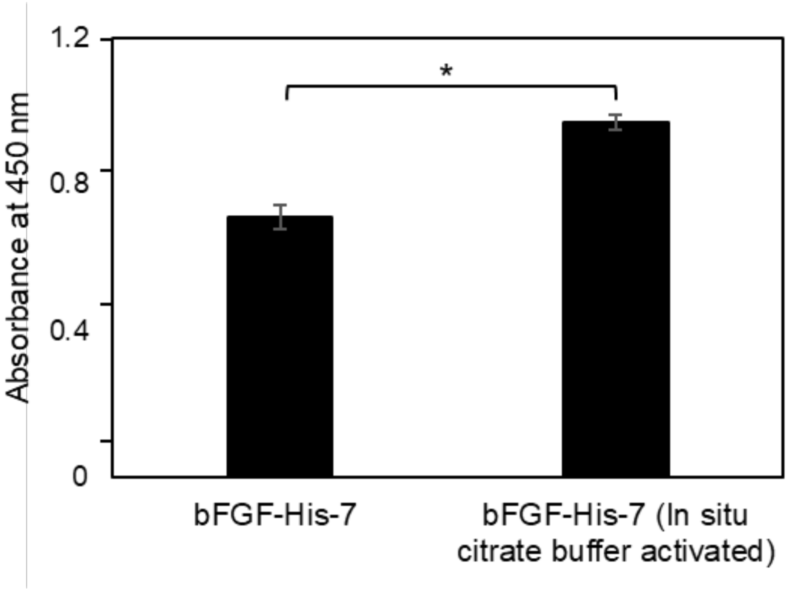

Figure S7. The effect of *in situ* citrate buffer treatment on the bioactivity of immobilized bFGF-His-7. The hMSC proliferation assays were performed 6 days after seeding. Data are expressed as the mean ± standard deviation for n = 4. Student's t-test was performed to analyze significance in difference between the two conditions (*$p < 0.05$).

Osteogenic differentiation.

Cells were seeded at 5,000 cells/cm^2 in 6-well plate in basal medium. Upon reaching 80% confluence, the medium was switched to osteogenic differentiation medium consisting of DMEM with low glucose (Sigma) supplemented with 10 % FBS (Hyclone), 1×10^{-7} M dexamethasone (Sigma), 10 mM β-glycerophosphate (Tokyo Chemical Industry Co., Ltd., Tokyo, Japan), 50 µg/ml ascorbate-2-phosphate (Sigma), 2 mM L-glutamine (Sigma), 100 U/mL of penicillin (Sigma), and 100 µg/mL of streptomycin (Sigma). The medium was exchanged every 3 days. On the 28th day of osteogenic induction, cells were fixed with 95% ethanol for 10 min, and then rinsed with water followed by exposure to 1% Alizarin-red S solution (Sigma-Aldrich) for 30 min. Finally, the cells were extensively washed with water, dried at room temperature, and observed under an optical microscope.

Chondrogenic differentiation.

Approximately 2.5×10^5 cells were centrifuged in a 15 mL conical tube at 500 *g* for 5 min to obtain a pellet. The pellets were cultured for 28 days in serum-free minimum essential medium eagle alpha modifications (αMEM, Sigma-Aldrich, St. Louis, MO, USA) containing 1×10^{-7} M dexamethasone (Sigma), 50 µg/mL L-ascorbic acid 2-phosphate (Sigma), 4.5 g/L D-glucose (Sigma), 100 µg/mL sodium pyruvate (Sigma), 1 % ITS$^+$ (BD Biosciences), 10 ng/mL of TGF-β3 (Peprotech, Rocky Hill, NJ), 2 mM L-glutamine (Sigma), 100 U/mL of penicillin (Sigma), and 100 µg/mL of streptomycin (Sigma). On day 28, the pellets were fixed with 4% paraformaldehyde for 15 min at 4 °C and then embedded in paraffin. Five-µm-thick sections were prepared, deparaffinized, and finally subjected to Alcian blue staining.

Adipogenic differentiation.

Cells were seeded at 2×10^4 cells/cm^2 in a 6-well plate and grown to above 80% confluence in basal medium. Thereafter, adipogenic differentiation was induced by subjecting confluent monolayers to 3–4 episodes of adipogenic treatments. Each episode had two steps; incubation with adipogenesis induction medium (DMEM with high glucose, 10% fetal bovine serum, 100 U/mL of penicillin, 100 µg/mL of streptomycin, 200 µM indomethacin, 1 µM dexamethasone, 500 µM 3-isobutyl-1-methylxanthine, and 10 µg/mL insulin) for 72 h and subsequent incubation with adipogenesis maintenance medium (DMEM with high glucose, 10% fetal bovine serum, 100 U/mL of penicillin, 100 µg/mL of streptomycin, and 10 µg/mL insulin) for 72 h. On day 28, the cells were fixed with 4% paraformaldehyde for 10 min, and lipid droplets formed in the cells were stained with 0.3% oil red O solution for 30 min.

References

1. Hirata, I. *et al.* Study of complement activation on well-defined surfaces using surface plasmon resonance. *Colloids Surf. B Biointerfaces* **18**, 285–292, https://doi.org/10.1016/S0927-7765(99)00154-X. (2000).
2. Paborsky, L. R., Dunn, K. E., Gibbs, C. S. & Dougherty, J. P. A Nickel Chelate Microtiter Plate Assay for Six Histidine-Containing Proteins. *Anal. Biochem.* **234**, 60–65, https://doi.org/10.1006/abio.1996.0050 (1996).
3. Knoll, W. Polymer thin films and interfaces characterized with evanescent light. *Makromol. Chem.* **192**, 2827-2856, https://doi.org/10.1002/macp.1991.021921201(1991).
4. Schmitt, J., Hess, H. & Stunnenberg, H. G. Affinity purification of histidine-tagged proteins. *Mol. Biol. Rep.* **18**, 223–230, https://doi.org/10.1007/BF01674434 (1993).
